# Loss of ‘Epidermal Melanin Unit’ Integrity in Human Skin During Melanoma-Genesis

**DOI:** 10.3389/fonc.2022.878336

**Published:** 2022-04-27

**Authors:** Cristina Casalou, Hugo Moreiras, Jay M. Mayatra, Aurelie Fabre, Desmond J. Tobin

**Affiliations:** ^1^ The Charles Institute of Dermatology, School of Medicine, University College Dublin, Dublin, Ireland; ^2^ Department of Histopathology, St Vincent’s University Hospital, Dublin, Ireland; ^3^ UCD School of Medicine, University College Dublin, Dublin, Ireland; ^4^ The Conway Institute of Biomedical and Biomolecular Science, University College Dublin, Dublin, Ireland

**Keywords:** melanogenesis, cutaneous melanoma, hair follicle, epidermal melanocytes, follicular melanocytes, vitiligo, hair follicle melanin unit, melanocyte-keratinocyte interaction

## Abstract

Cutaneous melanoma can be a most challenging neoplasm of high lethality, in part due to its extreme heterogeneity and characteristic aggressive and invasive nature. Indeed, its moniker ‘the great masquerader’ reflects that not all melanomas are created equal in terms of their originating cellular contexts, but also that melanoma cells in the malignant tumor can adopt a wide range of different cell states and variable organotropism. In this review, we focus on the early phases of melanomagenesis by discussing how the originating pigment cell of the melanocyte lineage can be influenced to embark on a wide range of tumor fates with distinctive microanatomical pathways. In particular, we assess how cells of the melanocyte lineage can differ by maturation status (stem cell; melanoblast; transiently amplifying cell; differentiated; post-mitotic; terminally-differentiated) as well as by micro-environmental niche (in the *stratum basale* of the epidermis; within skin appendages like hair follicle, eccrine gland, etc). We discuss how the above variable contexts may influence the susceptibility of the epidermal-melanin unit (EMU) to become unstable, which may presage cutaneous melanoma development. We also assess how unique features of follicular-melanin unit(s) (FMUs) can, by contrast, protect melanocytes from melanomagenesis. Lastly, we postulate how variable melanocyte fates in vitiligo, albinism, psoriasis, and alopecia areata may provide new insights into immune-/non immune-mediated outcomes for melanocytes in cutaneous melanin units.

## 1 Introduction

Skin cancer is the most common cancer in humans, with more cases diagnosed than for all other cancers combined (1). Although melanoma comprises less than 5% of all skin cancers, approximately 300,000 new cases are diagnosed globally/year. Melanoma remains a leading cause of cancer-related death (1, 2). The last 20 years have seen a tripling of melanoma diagnoses in the US, and now men less than 50 years of age have a higher chance of developing melanoma than any other cancer (1). If detected early, melanoma can be treated effectively by surgery, but as a metastatic disease, it can become highly resistant to conventional therapies. Until recently, treatment options for patients with advanced disease were very limited, but recent advances in both immuno- and targeted therapy have improved outcomes considerably by leveraging the patient’s own immune system against the tumor or targeting specific mutations (3). Still, melanoma is a malignancy with a poor prognosis, and improvements in overall patient survival have so far been limited; recent immunotherapy comes at a very high monetary cost (4). As a result, the current 5-year survival expectancy for patients with metastatic melanoma is approximately 23% (1, 5), with metastasis the leading cause of melanoma-associated deaths.

Cutaneous melanoma is a remarkably heterogeneous neoplasm, and clearly, not all melanomas are created equal. With its origin among melanocytes residing in the *stratum basale* of human epidermis, most cases of melanoma (>70%) appear to arise *de novo* without association with a pre-existing lesion (6, 7) e.g., a nevus. Invasive cutaneous melanomas are typically classified based on their clinico-histopathologic features into four main subtypes of decreasing incidence: superficial spreading melanoma (SSM), nodular melanoma (NM), lentigo maligna melanoma (LMM), and acral lentiginous melanoma (ALM) (8). Induction of a sustained proliferative potential in normally post-mitotic adult epidermis melanocytes results from mutations in genes predominantly of the MAPK pathway. Moreover, mutation signatures are different between melanoma subtypes (8, 9), and are often mutually exclusive. Melanoma mutations are common in *BRAF* (50%), *NRAS* (13.25%), *MEK1* (6%), and less commonly *KIT* (2.6%), *CTNNB*1 (2%–3%), *GNA11* (2%), or *GNAQ* (1%), highlighting the importance of controlled MAPK signaling for melanocyte homeostasis (9). Sini et al., recently contributed an interesting review concerning the genetic background responsible for melanomagenesis in human skin (10), reminding us that the main markers of melanoma are not necessarily responsible for the induction of the tumor. For example, BRAF, although is, indeed present (the mutated form) in 50% of melanomas, this BRAF mutation is also expressed in benign pigmented lesions (11).

While exposure to ultraviolet radiation (UVR) constitutes the major risk factor for cutaneous melanoma, and sun-protected melanocytes exhibit fewer mutations than sun-exposed ones, pigment cells from chronically sun-exposed skin (e.g., face) carry a lower mutational burden than melanocytes from intermittently-exposed skin (e.g., back). Thus, tumor progression is facilitated by a combination of genetic and epigenetic modifications, and people with pale skin, freckles, and red (pheomelanin-rich) hair exhibit the highest risk of melanocyte malignant transformation (12, 13). In addition to (epi)genetic characteristics, factors in the melanocyte microenvironment also modulate the oncogenic process e.g., extracellular matrix (ECM), microvasculature, intercellular communication (melanocyte-keratinocyte-fibroblast), as well as alterations in growth factors, cytokines, and nutrients. The formation of melanoma, therefore, shares essential characteristics with the variable stages in the life-history of melanocytes ranging from their development to maturation within human epidermis and hair follicle melanin units.

In this review, we restrict our focus to a discussion of our knowledge of the epidermal-melanin-unit (EMU) status in the skin and how alterations in melanocyte phenotype can disrupt their communication with their keratinocyte partners. Such disruption may lower the melanomagenesis threshold of these pigment cells.

## 2 Epidermal and Follicular Melanin Units of Normal Skin

### 2.1 Development of Cutaneous Melanin Units

**Figure 1:**
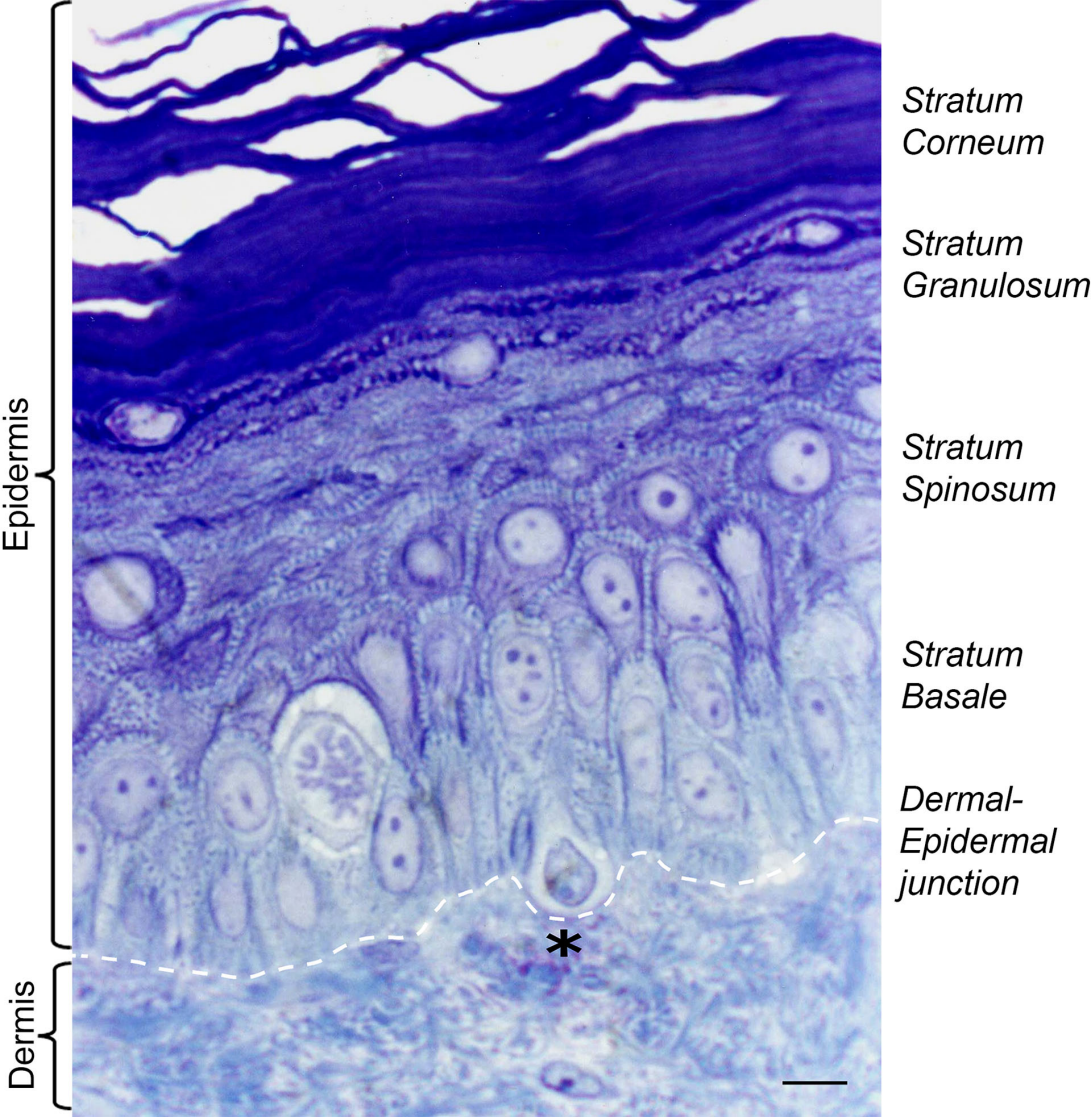
Histology of adult human skin. The epidermal layers of the skin are composed mostly of keratinocytes (90%) and melanocytes (3%). Melanocytes sit at the dermal epidermal junction, surrounded by proliferating keratinocytes of *stratum basale*, which differentiate as they reach the upper layers of epidermis. Black asterisk denotes a pendulous melanocyte localized at the basal layer of the human epidermis. Staining Toluidine blue (1%) in Borax solution (1%). Scale bar 5 μm.

Skin and hair pigmentation is the product of a remarkably complex communication between two histologically-distinct cell types; the pigment-producing melanocytes of neural crest origin and the pigment-accepting keratinocytes of epithelial origin. The interaction of one melanocyte with a highly stable number of keratinocytes generates a range of functional pigment or melanin units with unique features, which contribute to the homeodynamic balance of the epidermis and hair follicle. Melanocytes constitute a minor cell population (~3%) in the adult human interfollicular epidermis (see **Figure 1**). Their most apparent trait is the production of a light-absorbing indole biopolymer called melanin, via a phylogenetically-ancient biochemical pathway called ‘melanogenesis’ (14, 15). Apart from this primary role, melanocytes play important regulatory functions in skin, as potential contributors to skin immune response, stress sensors or as neuroendocrine cells, through the production of neuropeptides, neurohormones and neurotransmitters (16–18). For example, as a near-permanent cell in our epidermis, melanocytes act as ‘sentinel’ cells, through stress sensing and immune response modulation, especially in the context of UVR. While cutaneous melanocytes of the interfollicular epidermis are essentially post-mitotic cells, even with (UVR) stimulation, their cousins in the cycling hair follicle (see below) exhibit considerable plasticity, including in their proliferative capacity.

Melanin synthesis is a product of a series of enzymatic reactions, commencing with the hydroxylation of phenylalanine followed by the rate-limiting conversion of tyrosine by tyrosinase to form brown-black eumelanin and in the presence of cysteine or glutathione, the yellow-red pheomelanin, in the unique organelle of melanocytes - the melanosome (14, 15). In brief, melanogenesis involves a series of signals beginning with the rate-limiting oxidation of L-tyrosine to L-dihydroxyphenylalanine (L-DOPA), catalysed by tyrosinase (19, 20). Besides their positive regulatory role of melanogenesis, L-tyrosine and L-DOPA can act also as hormone-like regulators of other cellular functions. Upon stimulation by alpha-melanocyte-stimulating hormone (α-MSH) or adrenocorticotropic hormone (ACTH), melanocortin-1 receptor (MC1R) signaling can increase intracellular cyclic adenosine monophosphate (cAMP). The cAMP activates the response element-binding protein (CREB)-signaling pathway in melanocytes, to transcriptionally activate a variety of downstream targets, including microphthalmia-associated transcription factor (MITF). This crucial transcription factor activates essential melanin-forming genes such as the tyrosinase enzyme family and the melanosome matrix protein PMEL (14). Once melanogenesis is completed, eu-melanosomes assume an ellipsoidal form, while pheo-melanosomes retain their spherical shape with much lower structural organization (21). Mature melanosomes are transported (via multiple different modes) along dendrites and filopodia on their way to neighboring keratinocytes (22–25). This process represents an extremely rare example of organelle ‘donation’ from the cell type that makes it (i.e., melanocyte) to a wholly different histologic cell type (keratinocyte) that receives it. In the epidermis, melanin granules tend to aggregate as supranuclear caps within (often) proliferative keratinocytes, where it can protect their nuclear DNA from UVR-induced damage (26).

**Figure 2:**
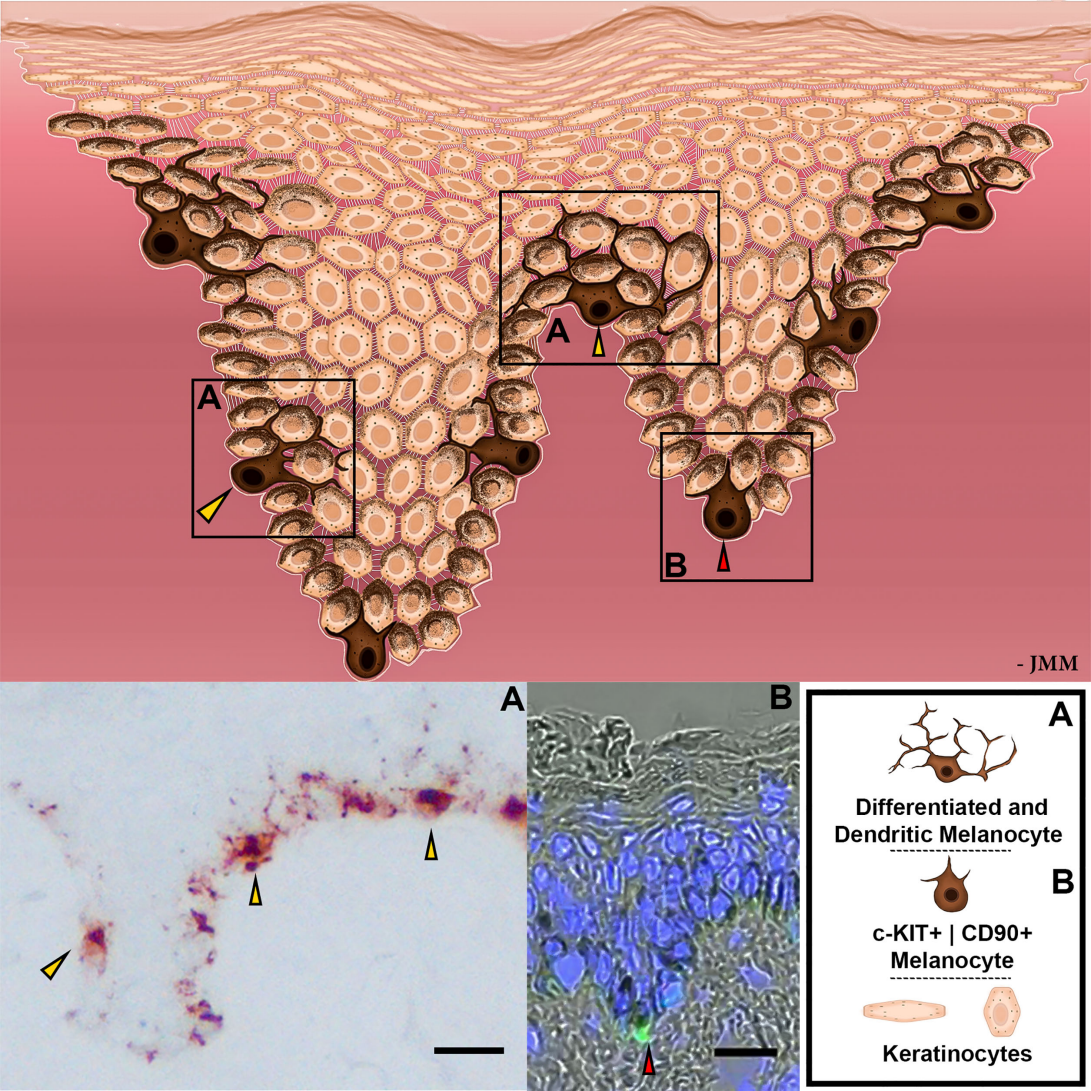
Schematic representation of EMUs. Distinct subpopulations of melanocytes are represented by different levels of differentiation, dendricity, melanin production, and cell receptor expression. Magnified images with differential melanocyte localization within the basal layer of human epidermis exhibited with gp100 (pre-melanosome antigens as detected by NKI/beteb antibody, #AB34165) immunostaining **(A)** or with c-KIT (in green; #orb178436) by IHC **(B)**. Nuclear staining is indicated by DAPI, in blue. Scale bar 20 μm.

Melanocytes and keratinocytes exist in the human epidermis within a truly extraordinary and remarkably-stable unit of one post-mitotic melanocyte to approximately 36 viable keratinocytes - the so-called ‘epidermal melanin unit’ or ‘EMU,’ as first proposed by Breathnach and Fitzpatrick in 1963 (27). This unit is crucial for maintaining the integrity of the human epidermis and especially for its protective skin pigmentation (see **Figure 2**). Given that all human skin phototypes, from black to white, have the same melanocyte number per unit area of epidermis, much of the qualitative variation in human skin color is due to differences in a) the number of melanin granules per melanocyte; b) melanin type (i.e., eu-/pheo-melanin), and c) pattern of melanin granule distribution throughout various *strata* of the human epidermis (27). Within the EMU, the keratinocyte partner tightly controls several aspects of melanocyte behavior, including *via* a regulated balance of paracrine growth factors and cell-cell adhesion molecules. Distinct subpopulations of melanocytes seem to be differentially positioned within the basal layer of the human epidermis, generating at least two EMUs (28, 29) (see below).

**Figure 3:**
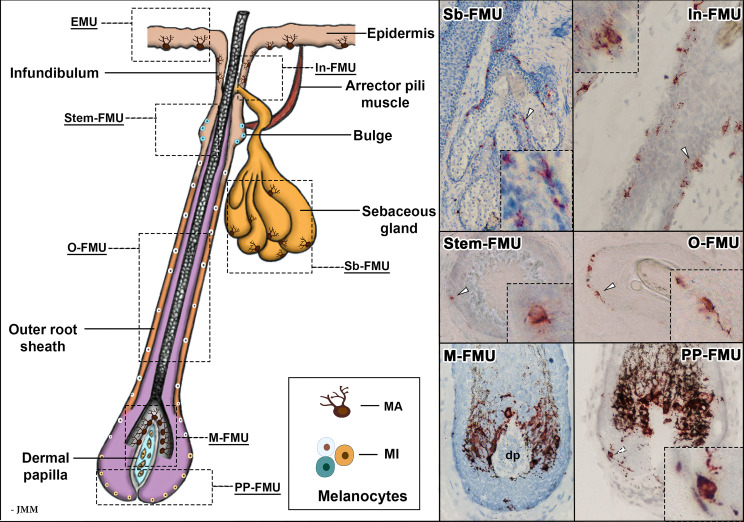
Schematic representation of an anagen VI hair follicle, showing the FMUs within the distinct compartments. Magnified images of melanocyte localization within six FMUs, exhibited with gp100 (pre-melanosome antigens as detected by NKI/beteb antibody, #AB34165) immunostaining. In-FMU, infundibulum follicular melanin unit; M-FMU, melanogenically-active follicular melanin unit; Sb-FMU, sebaceous gland follicular melanin unit; O-FMU, outer root sheath follicular melanin unit; PP-FMU, peripheral proximal bulb follicular melanin unit; Stem-FMU, stem cell niche follicular melanin unit; MA – melanogenically-active melanocytes: MI – melanogenically-inactive melanocytes; dp – dermal papilla.

Beyond the epidermis, the skin’s main appendage, the human hair follicle, contains a remarkable diversity of melanocyte subpopulations, the most apparent of which form a melanogenicially-active “follicular melanin unit” or “M-FMU” (30). These melanocytes, which engage principally with keratinocytes of the hair follicle pre-cortex, are located in the non-immunocompetent proximal anagen (growing phase) hair bulb and attach their cell bodies to the basal lamina separating the hair bulb epithelium from the mesenchymal (hair growth-inductive) follicular papilla (see **Figure 3**). Pre-cortical keratinocytes receive melanin from these M-FMU melanocytes to pigment the hair shaft (21, 30, 31). There also exist several other FMUs in anagen terminal (coarse) hair follicles, like those of the human scalp, operationally categorized on the basis of their melanogenic activity (see below). These include a) melanogenically-active melanocytes in the hair follicle infundibulum (In-FMU) and *S*ebaceous gland (Sb-FMU), and b) melanogenicially-inactive melanocytes located in the *S*tem cell niche of the hair bulge (Stem-FMU), Outer root sheath (O-FMU), and most Peripheral-Proximal hair bulb (PP-FMU) (See **Figure 3**).

Despite their shared neural crest origin and transient co-location in developing human epidermis [prior to the commencement of hair follicle morphogenesis (32)], clinical observations provide ample evidence for the relative independence of the EMUs and FMUs, especially in terms of how their respective melanogenesis is regulated. One can readily appreciate this in the striking snow-white hair of aged Black-Africans or the jet-black hair of alabaster, white-skinned youths of northwest European ancestry. Furthermore, there is selective and/or preferential EMU targeting in most cases of vitiligo, while M-FMU melanocytes alone are damaged by an immune-mediated pathology in acute alopecia areata (33). While some of these differences likely reflect distinct relationships with UVR (i.e., by virtue of their different locations in the skin), others are likely to involve differences in their respective immunological contexts and antigenic profiles *in situ*. The latter reflects, at least in part, the fact that FMUs in the anagen outer root sheath and hair bulb reside in a so-called ‘immune-privileged’ (MHC-I negative) part of the hair follicle epithelium (34, 35), contrasting with the immunocompetent MHC-I positive EMU. Interestingly, several of these melanocyte subpopulation differences can be retained in long-term culture (36, 37)

The mouse has been instrumental in providing significant insights into the regulation of mammalian pigmentation and, by extension, melanoma. However, readers should interpret these data with some caution, at least in the context of understanding how melanocyte-keratinocyte interactions in the epidermis are organized. This is so because mouse melanocytes are not present at the dermal-epidermal junction throughout most of their skin (except for ear, tail, nose, and food-pad), and mice do not develop melanoma without genetic manipulation. Moreover, induced melanomas in mice originate in the dermis, and while it is assumed that these tumors are derived from the proximal hair follicle, dermal melanocytes may also be a source of cutaneous melanoma in mice (38). Regardless, the very different melanocyte microenvironments in these two mammalian species will account for some of the many differences in melanoma development and progression between them. Furthermore, the heterogeneity of melanoma in outbred humans cannot be recapitulated in inbred laboratory mice, nor can the drivers for spontaneous melanoma development in humans. Indeed, the focus of mouse cutaneous pigmentation studies has been limited, in the main, to their hair follicle melanin unit (38).

#### 2.1.1 Epidermal Melanocyte-Keratinocyte Communication - the EMU

In the human epidermis, melanocytes produce unique subcellular lysosome-related organelles called melanosomes that are responsible for melanogenesis (24, 25). After melanin synthesis, each melanocyte extends multiple dendrites (and still finer filopodia) as cytoplasmic extensions (22) that ramify between approximately 36 viable keratinocytes (i.e., those located below the stratum granulosum), thereby forming the EMU (**Figure 2**) (27). These cytoplasmic connections provide conduits for the transfer of pigment granules (and likely other melanocytic cellular material) to both basal and suprabasal keratinocytes (22, 24, 25). In this way, melanocyte ‘donations’ to keratinocytes influences their physiology, not least by protecting this critical proliferative cell population from UVR damage (39). Epidermal homeostasis is maintained, therefore, by proliferation and subsequent differentiation of these melanin-containing keratinocytes as they migrate distally, retaining some continued bi-directional communication with their EMU partner melanocyte.

At least two subpopulations of melanocytes can be detected in the human epidermis, represented by different levels of differentiation, dendricity, activity of melanogenesis-related apparatus, and cell receptor expression (28, 29). These melanocyte subpopulations appear to occupy different anatomical locations within the epidermis, with c-KIT^+^ | CD90^+^ melanocytes more commonly distributed around the base of rete ridges (40) and more differentiated and dendritic melanocytes located more superficially, including in the inter-rete ridge epidermis (**Figure 2**). Whether melanocytes of these two EMU are similarly susceptible to melanomagenesis awaits further study (22). The stem cell factor (SCF)/c-KIT pathway is particularly important in the organization of the EMU in adult human skin, as it controls melanocyte proliferation and differentiation (41). This signaling pair may also play a role in melanogenesis, depending on whether the c-KIT ligand is membrane-bound (m-KIT) or soluble KIT (s-KIT) (42, 43). Interestingly, the binding of its ligand (SCF) to m-KIT is reported to stimulate melanogenesis, while s-KIT suppresses melanogenesis. Thus, UVR increases both SCF and m-KIT while decreasing s-KIT expression, thereby upregulating melanin synthesis (43).

This melanocyte duality within the epidermis may be more evident in younger skin, where there is extensive rete ridge plication. We postulate, therefore, that flattening of epidermis rete ridges in aged skin will likely impact how these melanocyte subpopulations are sustained. There is also likely to be a significant difference in the impact of UVB on more differentiated melanocytes located in the superficial inter-rete ridge epidermis versus less differentiated rete ridge crypt melanocytes, which can be located up to 200 µm deeper in the skin (40).

Melanin distribution in the human epidermis affords localized protection against DNA photodamage and, importantly, concurs with differences in the incidence of skin cancer seen across the range of human skin phototypes (37). For example, the observed 20- to 60-fold difference in keratinocyte cancers between white and black populations is largely attributable to high levels of epidermal melanin photoprotection in the latter. Most studies suggest that melanin photoprotection against cyclobutane pyrimidine dimers (CPD) (most mutagenic DNA photo-lesion) is modest and so cannot on its own explain the considerable skin color-based differences in skin cancer incidence. Melanin is most concentrated in the basal layer of skin and explains, at least in part, the considerable skin color differences in keratinocyte cancer incidence. Melanin in black skin protects against CPD formation by 59.0-, 16.5-, and 5.0-fold in the basal, middle, and upper epidermis, respectively (37), and so is related to the distribution of melanin. A greater appreciation of the regulation of eumelanogenesis versus pheomelanogenesis (44), in addition to precise melanin granule localization within *strata* of the human epidermis, is needed to leverage more optimal control of melanocyte/keratinocyte interactions in the EMU.

Despite melanin’s unambiguous optical properties at the skin surface, for at least the more melanoma-prone Caucasian populations, unstained melanin granules are only barely detectable outside the basal/suprabasal layer of the human epidermis. The apparent ‘absence/disappearance’ of melanin from suprabasal layers of the human epidermis has been interpreted, despite convincing evidence, as melanin dissolution/degradation within stratifying keratinocytes i.e., in the mid-to-upper epidermis. We have recently reassessed this view and provided evidence for a preferred ‘asymmetric inheritance’ of melanin granules by just one of the daughter cells following mitosis of progenitor keratinocytes in the basal layer (45, 46) i.e., the non-differentiating, self-renewable daughter cell. In this way, most melanin is retained in UVR-vulnerable and highly proliferative (i.e., progenitor) basal keratinocytes. One further interesting implication of this asymmetric mode of melanin inheritance by progenitor keratinocytes is that much lower levels of redox-sensitive melanogenesis may be needed than previously thought, as at least some pre-made melanin may be available for reuse within the basal layer of the epidermis. Interestingly, this baseline or default asymmetric pattern of melanin distribution can switch to a symmetric one under conditions of stress (i.e., wound-healing/regeneration/high UVR incidence), whereby both keratinocyte daughter cells now inherit similar levels of melanin cargo. In this way, the melanin level within the EMU can be modulated to respond to changing micro-environment and tissue demands.

#### 2.1.2 Follicular Melanocyte-Keratinocyte Communication – the FMU

Within the cutaneous family of pigment-producing cells, the second most prominent subpopulation of melanocytes resides in the hair follicle, especially during its growth (or anagen) phase of the hair cycle (47). The lives of pigment cells in this uniquely mammalian appendage are even more extraordinary and bewildering than those in the overlying epidermis. In human terminal hair follicles, follicular-melanin units “FMU”s are formed when one melanocyte interacts with a limited number of neighboring follicular keratinocytes (31). At least six distinct follicular melanocyte subpopulations can be detected in different compartments of the growing hair follicle and its associated sebaceous gland. These are grouped according to their respective phenotypic characteristics: stemness, melanin-production capacity, dendricity, and mitotic and apoptotic potential (**Figure 3**) (48). These include a) melanogenicially-active and dendritic melanocytes in hair follicle *I*nfundibulum (In-FMU) and *S*ebaceous gland (Sb-FMU); b) melanogenicially-inactive melanocytes in the hair bulge *S*tem cell niche (Stem-FMU), *O*uter root sheath (O-FMU); c) *M*elanogenicially-active melanocytes located adjacent to the hair follicle dermal papilla in the anagen hair bulb (“M-FMU”) and d) finally an enigmatic melanogenicially-inactive melanocyte subpopulation located in the most *P*eripheral-*P*roximal anagen bulb (PP-FMU) (see **Figure 3**).

Unlike melanocytes of the epidermis, the activity of follicular melanocytes is tightly coupled with the hair growth cycle (47). M-FMU pigmenting bulbar melanocytes direct melanin to progenitor pre-cortical keratinocytes (49), while the latter communicate with and instruct the melanocytes to become efficient pigment producers via transcription factors extracellular signaling proteins, and transmembrane receptors, etc. In this way, this cellular communication contributes to a homeostatic balance for optimal hair fiber pigmentation. M-FMU melanocytes exhibit several phenotypic differences from melanogenically-active melanocytes in the EMU. For example, M-FMU melanocytes produce 2- to 4-fold larger melanosomes and harbor more extensive dendrites (21) than differentiated melanocytes in the EMU.

The hair follicle is the only continually-cycling tissue throughout the lifespan of the mammal (47, 50). This growth cycle is a unique example of physiological deconstruction of a pigmentary unit (through programmed melanocyte death), followed by the reconstruction of the next melanin unit generation via a burst of melanocyte proliferation followed by cell maturation. Active melanogenesis within melanocytes and melanin transfer from melanocytes to keratinocytes occurs for up to 10 years or more in the human scalp) (51), suggesting that the melanogenic potential of a relatively small number of bulbar melanocytes in the M-FMU is much greater than that needed for the typical anagen length (around 3 years) of scalp hair follicle. We have previously reported that melanocytes of the M-FMU undergo selective deletion or cell death by apoptosis during a phase of the hair growth cycle, called catagen, involving the involution of ~70% of the hair follicle (28, 47, 48, 50). The catagen hair follicle then transitions to a phase of relative dormancy (telogen), only to be followed by a new anagen phase that is characterized by proliferation and differentiation of both melanocytes and keratinocytes.

## 3 EMU Disruption During Primary Melanomagenesis

### 3.1 Cutaneous Melanoma

Oncogenic transformation of a single melanocyte is the consequence of a multistage process of successive acquisition of (epi)genetic alterations affecting cell proliferation and survival (52). Like other solid malignant tumors, melanoma is characterized by uncontrolled proliferation, disruption of cellular and morphological differentiation, invasion, and metastatic spread to distinct organs. While different stages of melanoma progression are not easily distinguishable, pathological characteristics can be partially attributed to changes in intercellular communication between the transformed cell, its neighboring non-transformed cells, and their immediate microenvironment. One of the important early steps in melanoma development includes disruption of E-cadherin-mediated adhesive interaction between melanocytes and keratinocytes, accompanied by increased expression of N-cadherin, which facilitates proliferation and invasion of melanoma cells (53). Although changing interactions of melanoma cells with the tumor microenvironment are clearly very important, discussion of these phenotypic changes is beyond the scope of this review.

**Figure 4:**
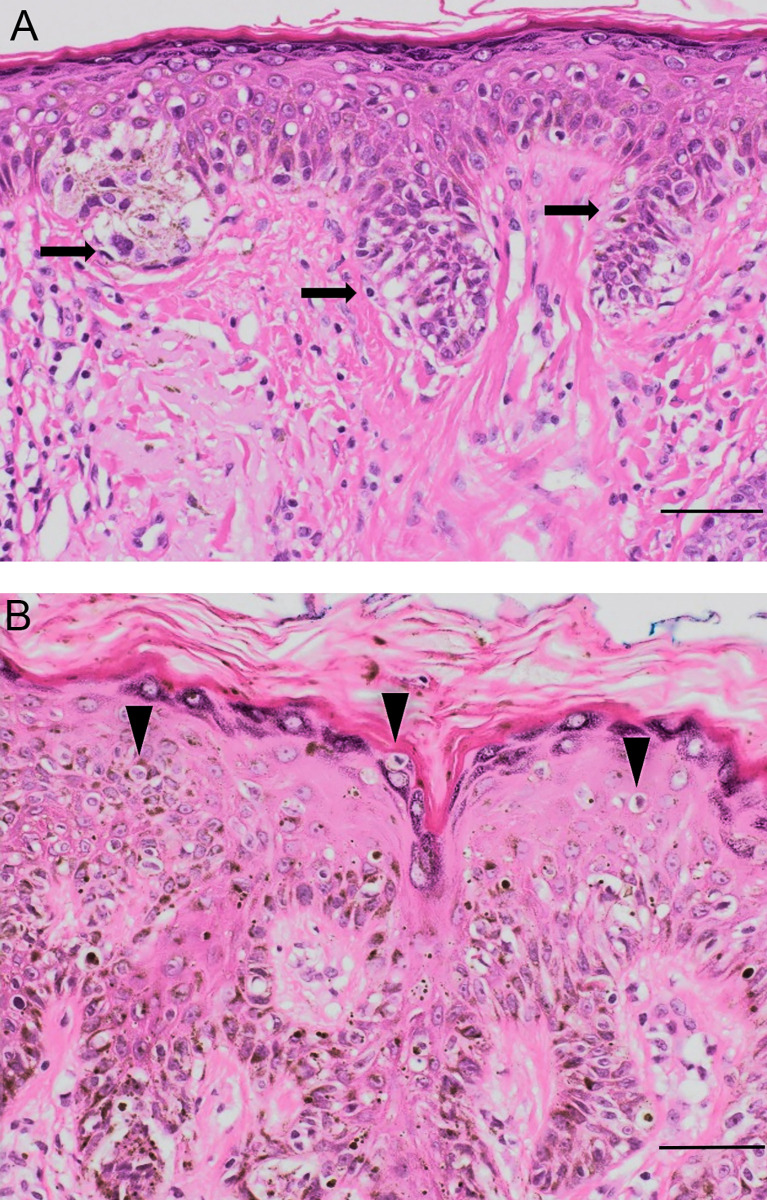
Pathological evidence of EMU disruption. **(A)** Dysplastic junctional naevus with nests and junctional mildly atypical melanocytes at the dermo-epidermal junction (black arrows). **(B)** Melanoma *in situ* with large melanocytes spreading upwards into the epidermis (arrowheads). Original magnification 50 μm, hematoxylin-eosin staining.

During oncogenic transformation, melanocytes in human skin begin to proliferate along the dermo-epidermal junction, progressively gaining independence from exogenous growth factors secreted by their keratinocyte partner cells in the EMU. Disruption in melanocyte-keratinocyte communication can trigger many phenotypic changes in both cell types, which may also include nesting into nevi and other benign lesions (54). With further damaging (genomic) alterations, single melanocytes or nevus nests of melanocytes may form dysplastic nevi or melanoma *in situ* subtypes (**Figure 4**).

Melanoma cells usually adopt an epithelioid shape and lack many features of their normal antecedents e.g., the melanocyte’s dendricity, lone distribution among 5-8 normal basal keratinocytes, and pendulous distribution into the upper papillary dermis whilst remaining firmly anchored to the basement membrane zone that separates the *stratum basale* of the epidermis and dermis (30). Evidence of EMU disruption can be observed in distinct histological subtypes of melanoma. SSM and LMM exhibit differences in their growth location and in the morphology of their melanosomal components. In melanomas with a superficial spreading component, a pattern of intra-epidermal growth occurs, often with a marked pagetoid (i.e., upward) spread of enlarged malignant cells (55). These can cluster at both the dermo-epidermal junction as well as more superficial epidermal areas of a now disorganized epidermis, characterized by the loss of the stable EMU cell ratio with keratinocytes (**Figure 4**).

On the other hand, the epidermis remains relatively thin in LMM, with proliferating malignant melanocytes restricted to the epidermal basal layer giving rise to a horizontal spreading phase referred to as Lentigo Maligna. There is typically other histologic evidence of severe solar damage (54) but only a modest increase in their EMU ratio with keratinocytes (**Figure 4**). By contrast, NM is characterized by a marked vertical growth pattern, whereby malignant cells exhibit dermal invasion accompanied by a higher mitotic rate compared to thin SSM (56). NM cells are often enlarged spindle or epithelioid shapes and can be organized into aggregates of so-called cerebroid nests (57, 58). As such, NM typifies a marked dysregulation of cell communication of the EMU, as well as with cells of the dermis.

Although the main function of melanin is to protect and buffer against UVR and redox imbalance, this pigment can also affect melanoma cell behavior. There is evidence of dysregulated melanin synthesis in the early stages of melanomagenesis (59), with melanin synthesis and subtype (eu/pheomelanin) correlated with melanosome morphology and integrity. Clinico-pathological analysis has shown that dysregulated melanogenesis can shorten the overall survival time (OST) and disease-free survival time (DFS) of patients with advanced metastatic melanomas (60). It has been shown that induction of melanogenesis in metastatic melanoma cells is closely linked to an increase of the hypoxia-inducible factor (HIF)-1α expression, with concomitant upregulation of HIF-dependent pathways involved in the regulation of glucose metabolism, angiogenesis and stress responses (61). In addition to altered melanization, dysregulation of the EMU has been associated with changes in melanosome matrix components (62). For instance, SSM melanosomes typically display a round-oval architecture and high pheomelanin content, while in LMM, melanosomes retain their usual ellipsoidal shape, typical of non-malignant melanocytes and are highly melanized (eumelanin) (60). NM, by contrast, are often hypopigmented or amelanotic tumors (63, 64). Indeed, there is growing interest in the potential role of pheo-melanogenesis in melanoma, both as a risk factor that reduces UVR-protection in the skin but also potentially due to some intrinsic (i.e., UVR-independent) pro-carcinogenic characteristic of the pheomelanin polymer itself (65). Earlier studies reported that dysplastic nevi synthesize more pheomelanin than eumelanin (66) and that this may be linked to dysregulated and chronic oxidative stress (67).

The involvement of keratinocytes in the crosstalk with malignant melanocytes has been reported. For example, aberrant suprabasal expression of keratin 14 in the epidermis surrounding NM has been detected i.e., a marker of associated keratinocyte hyperplasia (68). This study highlights the importance of the keratinocyte microenvironment in melanoma biology, particularly the necessity of a balanced communication within the cell subpopulations of the EMU. Further, rare examples of recurrent LMM co-mingling with nests of basal cell carcinoma have also been reported (69).

### 3.2 Connection Between Melanocyte Life Stages and Melanoma Progression

Phenotypic heterogeneity is a defining characteristic of melanoma, in part because melanoma cells can dynamically and reversibly switch between differentiated and undifferentiated states, thereby exhibiting distinct proliferative, invasive, and tumor-initiating characteristics (70, 71). Without a precise understanding of the true status and nature of the melanoma ‘originator’ cell(s) in human skin, it remains difficult to delineate how a defined population of normal melanocytes can initiate a transformation process that ultimately gives rise to such a heterogeneous tumor. It may be reasonable to assume that single cells within the tumor occupy distinct cell states (e.g., of differentiation) or different positions on a dynamic landscape (i.e., continuum). The proportion of cells in different states and positions may influence the outcome and prognosis of this neoplasm.

The literature contains a plethora of data supporting the origin of cutaneous melanoma from either melanocyte stem cells (McSCs) or from mature differentiated melanocytes. Confounding this issue is that most data has emerged from model organisms, particularly melanocytes of the mouse dermis, rather than from research focused on the pigmentation of human epidermis, nor even murine pigmented epidermis (ear/tail). This furred and nocturnal species lacks melanogenically-active melanocytes in their adult truncal epidermis and exhibits several other key differences, including the enzymatic regulation of melanogenesis (28).

During embryogenesis, committed melanocyte precursors or melanoblasts, originating from a multipotent neural crest (71, 72), (some perhaps also from Schwann cell precursors), migrate through the dermis from which they arrive to ‘invade’ the developing epidermis. Subsequently, a subpopulation of these melanocytes change from E-cadherin to P-cadherin-expressing cells, thereby enabling them to enter the developing P-cadherin-rich hair follicles as the latter start to develop (30). Although the melanin units of the epidermis and hair follicle are thereafter distinct, they do remain open, especially when the skin tissue is stimulated.

Still, it is important to emphasize that a considerable fraction of cutaneous melanocytes reside outside of the epidermis in adult human skin; mostly located in multiple distinct compartments of the hair follicle, its associated sebaceous gland, and even in sweat glands [see **Figure 3**; (73)]. Thus, the question of the precise location of the melanoma-targeted pigment cell must be very relevant in any discussion of human melanomagenesis. For example, upon stimulation (e.g., UVR or wound healing), hair follicle McSCs can indeed migrate to the interfollicular epidermis and differentiate into pigment-producing melanocytes (73–75).

But the hair follicle and sebaceous gland are not the only sources of melanocytes in the interfollicular epidermis post-stimulation. For example, studies from mouse tail skin lacking appendages show that this epidermis contains a low frequency of immature amelanotic melanocytes, which may function as interfollicular McSCs (76–78). These cells are reminiscent of the rare immature melanocytes that survive in the leucodermic epidermis of long-duration human vitiligo (79) or of immature melanocytes in hair-less glabrous skin - the suggested source for repigmenting of the lip in vitiligo.

However, available data suggest that melanoma may not arise from immature melanocytes, melanoblasts, etc. In fact, recent lineage-tracing studies using mouse tail epidermis (containing melanocytes) reported that melanoma arises instead from mature melanogenically-active melanocytes of the *inter-*follicular epidermis (80). These fully-differentiated cells appear to be reprogrammed and thereafter de-differentiate into cancer-initiating cells *in vivo*. Thus, differentiated pigment cells may acquire multipotency like their embryonic ancestors, allowing them to form heterogeneous tumors.

Other data supporting a melanocyte dedifferentiation mode of melanomagenesis is the coincident loss of melanocyte differentiation markers during melanoma progression, as well as an upregulation of genes associated with earlier stages of their development (81). The well-accepted concept in cancer biology that tumorigenesis recapitulates embryonic development has also been validated in a zebrafish melanoma model, in which loss of melanocyte signatures and emergence of neural crest signatures precede the expansion of melanoma (82). The relevance of this in public sun care policy may be important given the common ‘mole-watch’ focus of many melanoma prevention strategies, while at the same time, most melanomas (>70%) arise not from such lesions but rather arise *de novo*. This latter finding was given a powerful explanation from a human study where melanocytes in healthy skin were found to commonly contain pathogenic mutations (albeit weakly oncogenic ones) (83). This probably explains why they do not give rise to discernible melanocytic lesions. It also highlights that elucidation of the genomic ‘landscapes’ of individual melanocytes can provide insights into the cause and origin of melanoma. Increasing awareness of the clinicopathological and body-site differences between melanomas arising *de novo* or in association with a pre-existing pigmentary lesion supports the divergent pathway model of melanomagenesis (84).

While much of the above data is derived from modified mice models and requires urgent confirmation in human melanoma, these data suggest overall that melanoma can arise either from immature or mature pigment cells. This may explain the variability in cutaneous melanoma presentation and their outcomes. Therefore, it may be reasonable to suggest that the very significant heterogeneity observed in melanoma results from the involvement of several distinct subpopulations of melanocytes in different stages of their life histories and within various distinct cutaneous melanin units.

### 3.3 Does Hair Follicle-Derived Melanoma Exist?

A hallmark of the comparative biology of epidermal and hair follicle melanocytes is the observation that melanogenesis in the latter is stringently coupled to the hair growth cycle (anagen-specific melanogenesis), while melanogenesis in the former is continuous, albeit often augmentable (e.g., after exposure to UVR). Up to 90% of melanomas are said to be ‘caused’ by UVR from sunlight (85), and while much incident UVB and UVA readily reaches melanocytes in the superficial epidermis, hair follicle melanocytes are located more deeply in the skin, most located well below the level of UVB penetration (18, 29). That said, more superficially distributed melanocytes, e.g., in the upper infundibulum (In-FMU) and potentially also the bulge stem niche Stem-FMU), may receive carcinogenic doses of UVB. This may be particularly so in small hair follicles producing fine and vellus hairs (86).

However, it is remarkable that the skin’s main appendage, the hair follicle, appears to be largely resistant to melanomagenesis. This is despite the fact that keratinocyte neoplasms can occur in this skin appendage, although much less commonly than in the epidermis. If hair follicle tumors do occur, they are most often benign (e.g., pilomatrixoma); although some can be malignant (e.g., pilomatrix carcinoma) (86). This relative resistance of hair follicles to transform may be due to their inherent capacity to engage in life-long cycling (i.e., hair follicle cells naturally regenerate themselves). This may protect them against tumorigenesis and tumor growth, even in the presence of the well-known cancer-causing mutations (87). Melanomas do not typically arise from melanocyte/melanoblast subpopulations in the hair follicle, despite their life-long proliferative potential. Very rare exceptions, so-called follicular malignant melanoma, have been reported, including some arising from the sun-damaged skin of elderly individuals (88). However, even here, there is a strong possibility that this melanoma may not be primary in origin but rather folliculotropic i.e., migrate toward the hair follicle. Greater attention to these tumors may help us understand the origin of the melanoma cell, given that cycling hair follicles exhibit both the highest rate of epithelial cell proliferation (after the gut), as well as periodic bursts of melanoblast/melanocyte proliferation (28).

A contrary view has recently been proposed by Sun et al. These researchers genetically engineered a c-KIT promoter-driven mouse model so that these mice expressed oncogenic mutations (a combination of oncogenic *Braf* ^V600E^ induction and *Pten* loss) in their hair follicle McSCs (89). They were able to track their potential involvement in subsequent melanomagenesis, and found that these mutated melanoblasts, under the influence of Edn and Wnt signaling, migrated out of the hair follicle bulge into the overlying (melanocyte-free) epidermis and became invasive melanomas. Interestingly, these melanomas exhibited similar genetic and molecular characteristics to human melanomas. In another mouse melanoma model study, UVB-stimulated McSCs were translocated from the quiescent hair follicle niche into the epidermis in an inflammation-dependent manner (90). However, these studies are based on genetically-manipulated mouse models. It will need to be confirmed whether these are true models of human melanomagenesis.

Although the involvement of the upper hair follicle in spreading malignant melanoma has been reported, human melanoma characteristically progresses within the epidermis i.e., along the dermal-epidermal junction. This is in marked contrast to melanomagenesis in mice, which emerge from the dermis. In a study of 100 cases of primary human cutaneous melanomas, which also examined growth association with nearby hair follicles, most cases had melanoma tumor cells within at least one hair follicle. Of these, the vast majority of cases showed melanoma cells limited to the infundibulum . Less than a third of cases with hair follicle association showed melanoma cells extending a little deeper to the isthmus (the upper hair follicle between infundibulum and insertion site of arrector pili muscle). Remarkably, only in one exceptional case did the researchers detect melanoma cells below the level of the hair follicle bulge, located in the upper third of the hair follicle (91). These authors postulated that some ‘physiologic barrier’ might restrict the intra-epithelial spread of melanoma tumor cells at or beyond the level of the stem cell niche in the hair follicle bulge.

To our knowledge, clear evidence of melanoma originating solely within the M-FMU - the home of melanogenically-active and post-mitotic follicular melanocytes - is lacking (34). Together, these data suggest that the hair follicle contains a remarkably effective system of checks and balances, which may prevent melanocytes from going ‘feral’ (i.e., as in melanoma) or even from allowing migration of transformed pigment cells deep into the proliferative part of the growing hair follicle. The nature of such a physiological barrier remains unknown but may reflect the existence of an inhibitory ‘chalone,’ reminiscent of some such chalone that confers a ‘refractory’ state to some resting (telogen) hair follicles that fail to progress to a ‘competent’ growing stage during the hair growth cycle (92).

## 4 What Can Vitiligo, Albinism, Psoriasis, and Alopecia Areata Tell Us About Melanin Unit Integrity and Melanomagenesis?

Recently, we have become interested in the fate of melanocytes in several unrelated skin conditions, with and without an obvious pigmentary association. Remarkably, some skin disorders that are characterized by a disturbance in the integrity of their cutaneous melanin units (e.g., Vitiligo, Albinism, Psoriasis, Alopecia Areata) may be partially protected from melanomagenesis. This might come as a surprise to the reader after we have placed much emphasis above on the importance of maintaining melanin unit integrity to help prevent melanoma formation. Still, we believe much can be learned from these skin diseases, especially in how the melanocyte-keratinocyte symbiotic unit is regulated.

Since the advent of biologics, many melanoma patients treated with anti-PD-1/PD-L1 antibodies show signs of induced cell death of their normal epidermis melanocytes (93). This skin depigmentation has more than a passing resemblance to the presumptive autoimmune disorder vitiligo. In the former scenario, it appears to result from drug-associated induction of a cytotoxic T-cell mediated anti-melanoma immune response, where target antigens are expressed on both melanoma cells and normal melanocytes (e.g., MART-1, GP100, TRP1-2, tyrosinase). These observations suggest that melanocytes and derived melanoma cells share much immunologically, but also that cell death could possibly be leveraged across both normal melanocytes and melanoma, as seen in this treatment ‘spill over’ (94).

However, not all melanocytes are deleted in the leucodermic epidermis of vitiligo patients, even after several decades (79), suggesting that these melanocytes can somehow avoid immune-mediated detection or at least immune-mediated deletion. That leucodermic epidermis can be repigmented in glabrous (e.g., lip) as well as hairy skin suggests that not all repigmenting melanocytes need to emerge from the upper hair follicle (95, 96). Perhaps counter-intuitively, vitiligo epidermis is also remarkably resistant to actinic damage and skin cancer (97, 98). Patients with vitiligo may have 3-fold fewer skin cancers than their healthy partners. Importantly, this is true not only for keratinocyte cancers (squamous cell carcinoma and basal cell carcinoma) (99) but also for melanoma. Indeed, if skin cancer is ever detected in vitiligo patients, this is usually in their ‘normally pigmented skin (100). Fascinating work by Schallreuter and colleagues reported evidence for upregulated wild-type p53 together with p76 (MDM2); major players in the control of DNA damage/repair, suggesting their involvement in the prevention of photodamage and skin cancer in vitiligo (101).

Patients with the hypopigmentary disorder oculocutaneous albinism, which unlike vitiligo, presents a normal density of melanocytes in their cutaneous melanin units, are by contrast very susceptible to keratinocyte skin cancers. Some die of this complication. However, the occurrence of melanomas in South African Albino patients has been reported to be very rare, with most of the published studies reporting significantly less than 1% melanoma incidence in this patient group (102, 103). On the surface, this may appear paradoxical given that eumelanin is photoprotective, and its absence or reduction may be expected to increase the risk of UVR-associated DNA mutations characteristic of melanoma. As perhaps expected, when melanoma is found in these albino patients, it is usually amelanotic. There may be several issues at play here, however. Residual pigment or altered melanogenesis chemistry in these patients could represent a risk factor for amelanotic melanoma (104). However, melanoma incidence among Black South Africans is broadly like its incidence in Black South African albinos, also at lower than 1%, in contrast to a lifetime risk of 2.6% for melanoma in Caucasians (105). Thus, black skin’s protection from melanoma may not be due solely to an increased absolute amount of melanin pigment, but rather their melanocytes may have some additional (epigenetic) trait that confers them protection from melanoma, even in the absence/reduction of eumelanin.

Another interesting disorder where the integrity of the melanin unit is disturbed but where the skin also appears to be protected from melanomagenesis is the common hyper-proliferative dermatosis psoriasis (106, 107). We have recently reported that melanocytes can indeed proliferate in the epidermis of lesional and perilesional psoriasis (108). This was quite unexpected as melanocytes exist in a post-mitotic state in non-neoplastic skin. Epidermal melanocyte proliferation in psoriasis may be associated with the massive keratinocyte hyperplasia characteristic of psoriasis, which drives extreme elongation of epidermis rete ridges with a consequent massive expansion of the basal layer surface area wherein melanocytes reside. In that way, melanocyte proliferation may be a compensatory response to such rete ridge elongation (due to the keratinocyte hyperplasia) i.e., to maintain EMU’s stable melanocyte-keratinocyte ratio in the basal layer of the epidermis. It is also possible the psoriasis-associated immune-mediated stimulation of the epidermis may (transiently) shift melanocytes out of their usual post-/non-mitotic state. Regardless, it may be considered reasonable that proliferating melanocytes in a highly-inflamed psoriatic epidermis may exhibit an increased risk of progressing to melanoma. Remarkably such an increased risk of melanomagenesis does not appear to occur in these patients. This is even more remarkable as psoriasis patients often experience extended periods of PUVA (Psoralen and UVA) treatment (107). Treated psoriatic skin can exhibit increased numbers of senescent keratinocytes, which contribute to a field effect in the epidermis via secretion of cellular factors. This may help prevent tumor progression in adjacent cells. It would be interesting to explore the role of such cellular factors as anti-proliferative and anti-angiogenic factors in a pre-malignant human epidermis environment.

Lastly, alopecia areata, a non-scarring hair loss disorder, also shows several pigmentary anomalies. There is often a preferential targeting of pigmented hair and a relative sparing of white hair in these patients. While part of the FMU is selectively targeted in this disease [i.e., the M-FMU (31)], the rate of melanoma in these patients is reported to be low (109). Paradoxically, this observation may be linked to a melanocyte-targeted immune response in the alopecia areata-affected hair follicle (35). Indeed, it is postulated that localized hair depigmentation in alopecia, like vitiligo cases, could be an example of antigen-specific immunity in melanoma patients. In these patients, hair loss might be a side effect of T-lymphocyte-mediated cross-reactivity between tumor cells and protected melanocytes of the hair bulb (110). Clearly, a lot remains to be learned about the immunological response to melanocytes in different skin compartments and across different skin disorders.

## 5 Conclusions

The collective understanding of melanoma pathogenesis and genomics has expanded dramatically in the past few decades, leading to a marked increase in our knowledge of melanocyte biology and the identification of potential targets for treatments. Despite the significant progress in the management of this disease, melanoma still has a poor prognosis for many patients. Therefore, an in-depth awareness of the journey from the tumor originator cell (of the melanocytic lineage) to the malignant melanoma cell, and the selective pressures that operate on them during the early phases of transformation, is needed. This is due in part to the complexity of melanoma genetics involving variable gene-gene interactions and several immune escape mechanisms, but it is beyond doubt that altered cell-cell communication plays a critical role in melanoma development. With regards to melanomagenesis the interaction of the melanocytes and keratinocytes in the symbiotic relationship of the EMU is crucial to the maintenance of skin homeostasis.

In this review, we focus on the early phases of melanomagenesis, stepping back to consider the role of (un)stable melanin units (EMU) on melanocyte oncogenic transformation. Melanocytes of the human skin epidermis are highly resistant to cell-death by apoptosis, contrasting with melanogenically-active melanocytes in the hair follicle (FMU). There appear to be some protective mechanisms in the hair follicle that limit genomic instability and melanomagenesis.

In summary, the very significant heterogeneity of cutaneous melanoma tumors appears to derive from the existence of multiple melanocyte types. These may exist either as distinct cell subpopulations or as cells in different stages of melanocyte differentiation. Both are likely to relate directly to their location within the different skin melanin units. Greater knowledge of how to target unstable melanocytes may provide new avenues for melanoma prevention and treatment.

## Authors Contributions

Conceptualization – CC, DJT, HM, AF. Original draft and writing the manuscript – CC, DJT, HM. Writing section and reviewing spelling of the manuscript – JMM. Schematic design and preparation of figures – JMM, AF, CC. All authors read and agreed to the final version of the manuscript.

## Funding

Writing this review was supported in part by a grant to DJT from Science Foundation Ireland (SFI; 19/FFP/ 6752) and a Newman Fellowship, supported by CDSCHC & Janssen via the UCD Foundation, attributed to HM.

## Conflict of Interest

The authors declare that the research was conducted in the absence of any commercial or financial relationships that could be construed as a potential conflict of interest.

## Publisher’s Note

All claims expressed in this article are solely those of the authors and do not necessarily represent those of their affiliated organizations, or those of the publisher, the editors and the reviewers. Any product that may be evaluated in this article, or claim that may be made by its manufacturer, is not guaranteed or endorsed by the publisher.

## Abbreviations

UVR, ultraviolet radiation; UVA, ultraviolet radiation type A; UVB, ultraviolet radiation type B; SSM, superficial spreading melanoma; NM, nodular melanoma; LMM, lentigo maligna melanoma; ALM, acral lentiginous melanoma; HF, hair follicle; ECM, extracellular matrix; cyclobutane pyrimidine dimers, CPD; EMU, epidermal-melanin unit; FMU, follicular-melanin unit; McSCs, melanocyte stem cells; In-FMU, infundibulum follicular-melanin unit; M-FMU, melanogenically-active follicular melanin unit; Sb-FMU, sebaceous gland follicular melanin unit; O-FMU, outer root sheath follicular melanin unit; PP-FMU, peripheral proximal bulb follicular melanin unit; Stem-FMU, stem cell niche follicular melanin unit; SCF, stem cell factor; cAMP, cyclic adenosine monophosphate; MITF, microphthalmia-associated transcription factor; MC1R, melanocortin-1 receptor; α-MSH, alpha-melanocyte-stimulating hormone; MHC-I, major histocompatibility complex class I; DNA, deoxyribonucleic acid; CREB, cAMP response element-binding protein; PD1, Programmed cell death protein 1; PD-l1, programmed death-ligand 1; IHC, ImmunoHistoChemistry; L-dihydroxyphenylalanine, L-DOPA; L-dihydroxyphenylalanine, L-DOPA; hypoxia-inducible factor, HIF
